# Primary outcomes of combined cataract extraction technique with Ab-Interno trabeculectomy and endoscopic Cyclophotocoagulation in patients with primary open angle Glaucoma

**DOI:** 10.1186/s12886-020-01643-2

**Published:** 2020-10-09

**Authors:** Juan Carlos Izquierdo, Josefina Mejías, Laura Cañola-R, Natalia Agudelo, Bárbara Rubio

**Affiliations:** 1 Glaucoma Research Department, Instituto de Ojos Oftalmosalud, Lima, Peru; 2 Glaucoma Department, Instituto de Ojos Oftalmosalud, Lima, Peru; 3 Instituto de Ojos Oftalmosalud, Av. Javier Prado Este 1142, San Isidro, Lima, Peru

**Keywords:** MIGS, Endociclophotocoagulation, Micro-incisional glaucoma surgery, Trabecular meshwork, Kahook dual blade

## Abstract

**Background:**

Glaucoma surgery have been developed to lower intraocular pressure in a less invasive manner than traditional glaucoma surgery. The purpose of this article is to determine the outcome of using combined phacoemulsification technique, ab-interno trabeculectomy dual blade and endoscopic cyclophotocoagulation (ECP) surgeries in patients with primary open angle glaucoma.

**Methods:**

A retrospective case series was performed on 27 consecutive eyes with both primary open-angle glaucoma (POAG) and cataract; each eye was treated with combined phacoemulsification, ab-interno trabeculectomy-Kahook Dual Blade and Endocyclophotocoagulation at Instituto de ojos Oftalmosalud, Lima, Peru, between April 2017 and May 2017. Inclusion criteria: 1) Patients with uncontrolled mild to advanced POAG (according to Glaucoma Grading Scale HODAPP) 2) cataract condition 3) treatment with two or more glaucoma medications due to rapid progression in the visual fields (at least two in a short period of time). Intraocular pressure (IOP), best corrected visual acuity (BCVA) logMAR and number of glaucoma medications were recorded prior to the study, at day 1, week 1, and 1,3,6 and 9 months after surgery. Primary outcome measure was surgical success defined in terms of IOP < 14 mmHg either with no medications (complete success) or with medications (qualified success).

**Results:**

A total of 27 eyes from 27 patients were included. The mean basal IOP was 17.0 ± 3.7 mmHg and postoperatively was 11.6 ± 1.9 mmHg and 11.4 ± 1.8 mmHg (*P* < 0.001) at 6 and 9 months respectively. Glaucoma medications decreased from 1.9 ± 1.4 to 0.56 ± 1.05 at 9 month follow-ups (*P* < 0.001). Preoperative best corrected visual acuity (BCVA) showed and improvement from 0.4 ± 0.4 LogMAR to 0.2 ± 0.4 logMAR at 9 months. The main complication was blood reflux intra-operatively (66.7%), which resolved without re-operation. The mean IOP was reduced by 32.9% from baseline and the surgical success was 92,6%, (complete success 70,3% and qualified success 29,6%) at 9 months.

**Conclusions:**

In patients with POAG, combined treatment with phacoemulsification, ab-interno trabeculectomy and endoscopic cyclophotocoagulation effectively reduced IOP and glaucoma medication dependence.

## Background

In geriatrics, it is common to find in patients with both cataract and glaucoma, both of which can be treated with combined phacoemulsification and glaucoma surgery to improve vision and avoid progression while improving cost-effectiveness [1].

The pathophysiology of open angle glaucoma is described as resistance outflow of aqueous humour, which is mainly the juxtacanalicular portion of the trabecular meshwork (TM). By performing an ab-interno trabeculectomy, the disease portion is removed and the conventional outflow pathway is enhanced with posterior reduction of intraocular pressure (IOP) [2–5].

Endoscopic cyclophotocoagulation is one of the safest armamentarium therapies for glaucoma; the ciliary processes are visualized directly using diode laser energy treated precisely until shrinkage and whitening occur. This therapy causes a reduction of aqueous humour production which decreases IOP effectively without the complications described for cycloablative procedures such as persistent hypotony, phthisis, inflammation or visual loss [6, 7].

Gold standard filtering surgery is associated with high rate of immediate and late complications as reported in the TVT study. For this reason, there is a trend to treat glaucoma patients with minimally-invasive procedures when maximal tolerated medical therapy fails to control visual field loss in initial or moderate glaucoma.

In this study a combined treatment of Phacoemulfisication, ab-interno trabeculectomy (Kahook) and ECP was performed to treat uncontrolled POAG and double mechanism for reducing IOP was expected. An update of the technique, reduction of IOP, glaucoma medications and visual acuity were evaluated.

## Methods

This retrospective case series comprised 27 eyes of 27 patients, with uncontrolled open-angle glaucoma (POAG) and cataract, having combined phacoemulsification, ab-interno trabeculectomy with Kahook Dual Blade (KDB) (New World Medical Inc., Rancho Cucamonga, CA, USA) and ECP, at Instituto de ojos Oftalmosalud, Lima, Peru, between April 2017 and May 2017. The ab-interno trabeculectomy was performed 90–120 degrees, and endoscopic cyclophotocoagulation was performed 360 degrees through 2 site corneal incisions. The mean duration of the follow-up period was 9 months.

The study was approved by the Ethics Committee of the Instituto de Ojos Oftalmosalud, in accordance with the Declaration of Helsinki. All patients provided written informed consent forms prior to enrolment.

Inclusion Criteria: Glaucoma patients with uncontrolled POAG from mild to advanced, according to Glaucoma Grading Scale (HODAPP), cataract condition, treatment with two or more glaucoma medications; uncontrolled was defined as progression in at least 2 visual fields and/ or retinal nerve fibre layer thinning in spfectral domain optical coherence tomography (sdOCT),

Exclusion Criteria: history of glaucoma surgery, any subsequent glaucoma surgery in the follow-up period, narrow angles or closed angle glaucoma, neovascular, uveitic or other secondary glaucoma, retinal or neurophthalmic diseases.

The intraocular pressure (IOP), best corrected visual acuity (BCVA) LogMAR and number of glaucoma medications were recorded prior to treatment, at day 1, week 1, and 1, 3, 6 and 9 months after surgery. At each visit, the IOP was measured with a Goldmann applanation tonometer. The intra and postoperative complications were recorded. Success was defined as IOP < 14 mmHg with or without glaucoma medication.

### Surgical modified technique

All procedures were performed by the same surgeon (JCI). Firstly, phacoemulsification and IOL implantation was performed using 2,2 mm keratome and 1.20 mm side-port blade, the anterior chamber was filled with preservative-free lidocaine 1%, and an ophthalmic viscosurgical device (OVD) (Healon GV; Abbott Medical Optics, Santa Ana, CA, USA). Continuous curvilinear capsulorhexis (CCC) was created with a capsulorhexis fórceps and lens segmentation was performed by using a stop and chop technique. Surgery was completed by implantation of an intraocular lens (IOL) in the capsular bag after successful removal of the lens cortex.

**Fig. 1:**
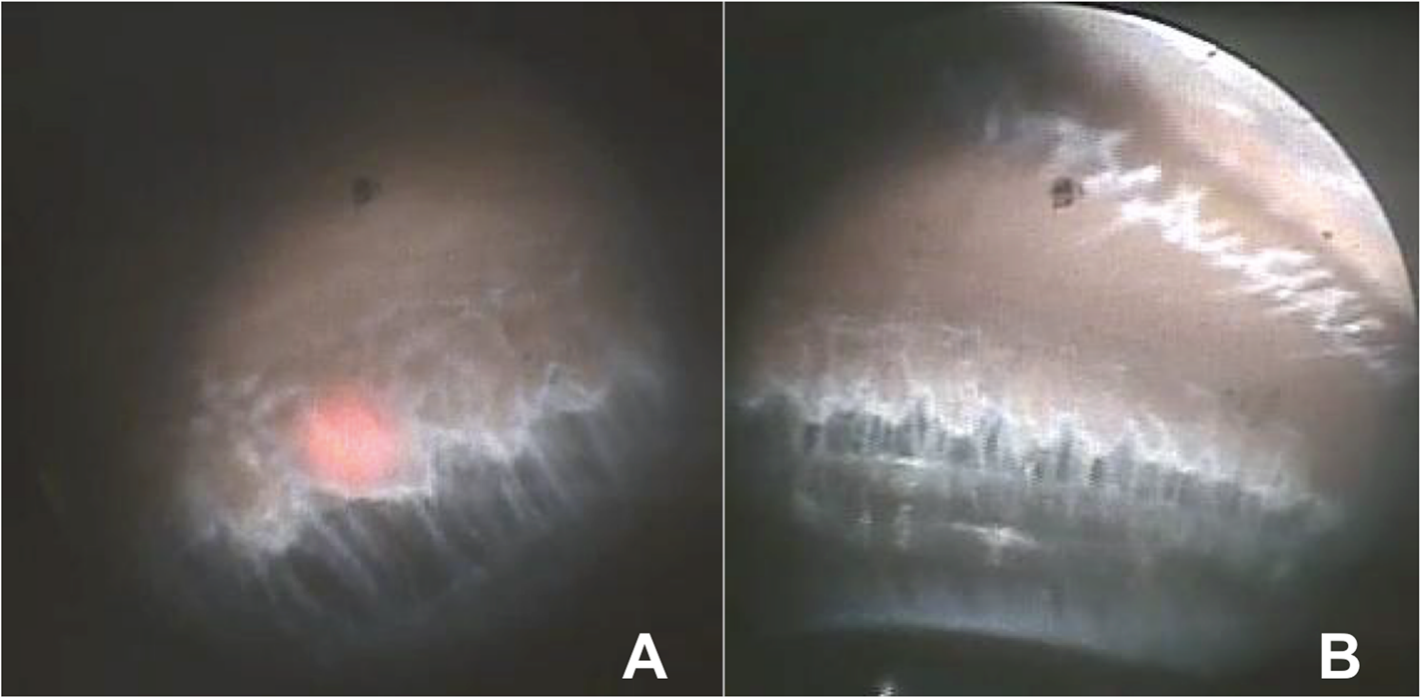
Endocyclophotocoagulation. Ablation of the ciliary processes. The physical goals of treatment were to whiten the ciliary processes and cause visible shrinkage of the tissue, avoiding rupture

Secondly, ECP (Endo Optiks® E2 Ophthalmic Laser Endoscopy System, Beaver-Visitec International, Inc. USA) containing an endoscope, an illumination source, the diode LASER (810 nm) and a helium-neon aiming beam, was inserted into the anterior chamber via the main corneal incision. Once the intraocular lens (IOL) was positioned in the bag, viscoelastic was injected to expand the sulcus behind the iris, and the diode laser ablation of the ciliary processes was performed under direct visualization via a 20G endoscope. In order to reach 360 degrees of the ciliary processes, a second incision 180 degrees away from the initial incision was made. The physical goals of treatment were to whiten the ciliary processes and cause visible shrinkage of the tissue, avoiding rupture. The 2-site corneal incisions 360 degrees were treated successfully with 0.2 W energy on continues mode (Fig. 1).

**Fig. 2:**
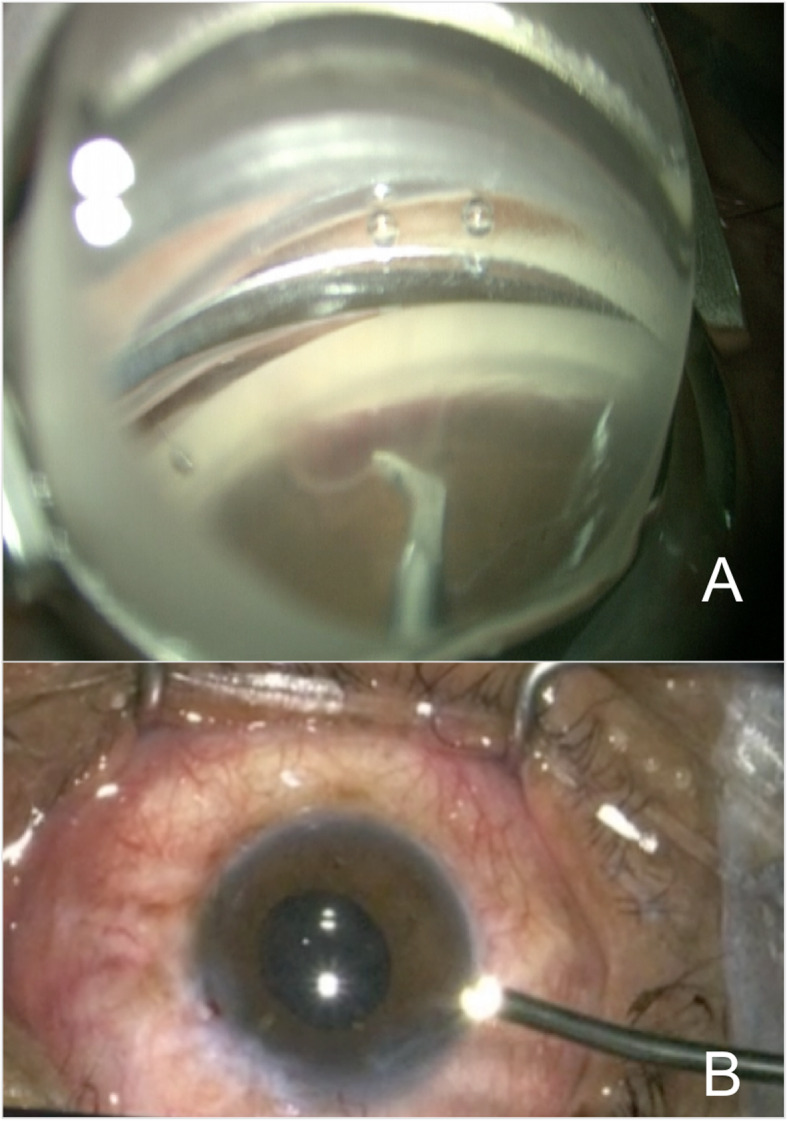
Steps of the surgery. **a**.- The Kahook dual-blade was engaged in the TM and was advanced 90 degrees parallel toward the wall of the canal. **b**.- Secondly, ECP containing an endoscope, an illumination source, the diode LASER (810 nm) and a helium-neon aiming beam, was inserted into the anterior chamber via the main corneal incision

Finally, a 2.2 mm wide iris planar clear corneal incision is fashioned approximately 2 mm anterior to the surgical limbus and viscoelastic was used in the anterior chamber. The patient’s head was rotated 40 degrees away from the surgeon and the microscope was tilted in the opposite direction for gonioscopic visualization, with the goniolens (AVG; Surgical Gonio Lens, Volk Alcon, Mentor, OH, USA). Trypan blue was use to stain the TM. The Kahook dual-blade (KDB; New World Medical, Rancho Cucamonga, CA, USA) was engaged in the TM just anterior to the scleral spur for a more pointed entry into the meshwork and was advanced 90 degrees parallel toward the wall of the canal. Intracameral dexamethasone 0.8 mg/0.2 ml was administered to all patients in addition to standard cataract antibiotic prophylaxis (intracameral cefuroxime 1.0 mg/0.1 ml). The corneal incisions were then closed with 10–0 nylon (Fig. 2).

Postoperative care routinely included tobramycin 3 mg/ml and dexamethasone 1 mg/ml (Trazidex, Sophia, México) 6 times daily tapered over 1 week, and pilocarpine 1% (Pil, Sophia, México) 3 to 4 times daily tapered over 4 weeks. In all cases, the glaucoma medication was discontinued at the time of surgery and restarted in selected cases according to IOP.

### Statistical analysis

To compare the changes in IOP according to the observation period, the non-parametric Friedman test was used, followed by a post-hoc Nemenyi multiple comparison test. For change in the number of glaucoma medications, the signed test for paired samples was used. Statistical tests we considered significant if *p* value was less than 0.05. The analysis was done with statistical software R, versión 3.4.3 (https://www.r-project.org/).

## Results

This study included 27 eyes from 27 patients who underwent the combined surgical procedure and were followed for at least 9 months. Mean age was 69.1 ± 8.09 (range: 53–85) years old. A total of 16 females (59.2%) and 11 males (40.7%), 10 (37%) right eyes and 17 (62.9%) left eyes were analysed.

**Fig. 3:**
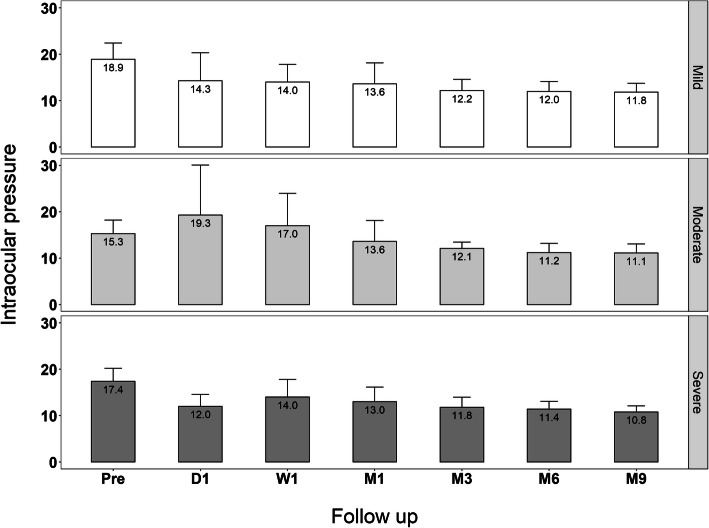
Comparison of IOP vs Follow-up time. All eyes with POAG were classified into 3 groups: mild, moderate and advanced. At 9 months, IOP reduction was 7.1 mmHg in the mild group, 4.2 mmHg in the moderate glaucoma group and 6.6 mmHg in the advanced glaucoma

All eyes with POAG were classified into 3 groups according to Glaucoma Grading Scale (HODAPP): 10 (37%) eyes were mild, 10 (37%) eyes moderate, and 7 (25.9%) eyes advanced. All 3 categories had a reduction in IOP at 9 month follow-ups; however, multiple comparisons with Nemenyi test after Friedman test showed a statistically significant improvement at 3 months (*p* = 0,010) in the mild glaucoma group, at 9 months for the moderate glaucoma group (*p* = 0.031) and without statistically significant improvement in the advanced glaucoma group in any follow-up time, which can be attributed for the smaller sample size of this group. At 9 months, IOP reduction was 7.1 mmHg in the mild group, 4.2 mmHg in the moderate glaucoma group and 6.6 mmHg in the advanced glaucoma. (Fig. 3).

### Intraocular pressure

**Fig. 4:**
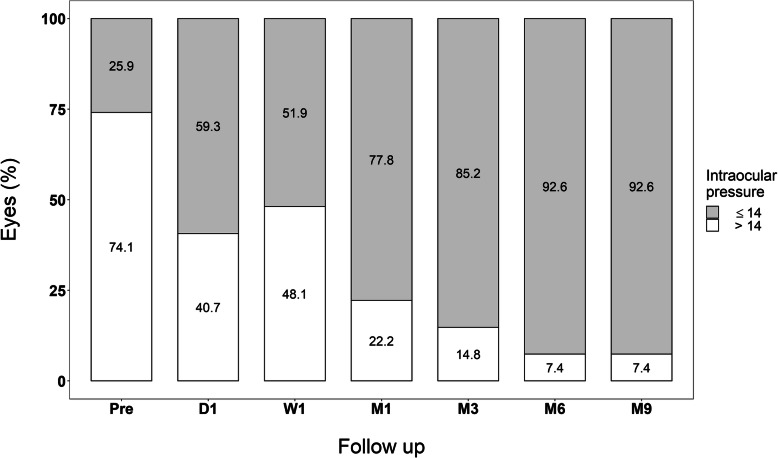
Percentage of eyes with Intraocular Pressure under 14 mmHg. The mean preoperative IOP was 17.0 mmHg [± 3.7]. and the postoperative IOP were 15.0 mmHg [± 5.3], 13.4 mmHg [± 4.1], 12.1 mmHg [±1.9], 11.6 mmHg [± 1.9.], 11.4 mmHg [± 1.8] at 1 week, 1, 3, 6, and 9 months respectively, (*p* < 0.001)

The mean preoperative IOP was 17.0 mmHg [± 3.7] and the postoperative IOP were 15.0 mmHg [± 5.3], 13.4 mmHg [± 4.1], 12.1 mmHg [±1.9], 11.6 mmHg [± 1.9.], 11.4 mmHg [± 1.8] at 1 week, 1, 3, 6, and 9 months respectively, (*p* < 0.001). The mean IOP was reduced by 32.9% from baseline and the success was 77.8% in the first month and 92.6% (complete success 70,3% and qualified success 29,6%) at 9 month follow-ups. (Fig. 4).

### Medication outcomes

**Fig. 5:**
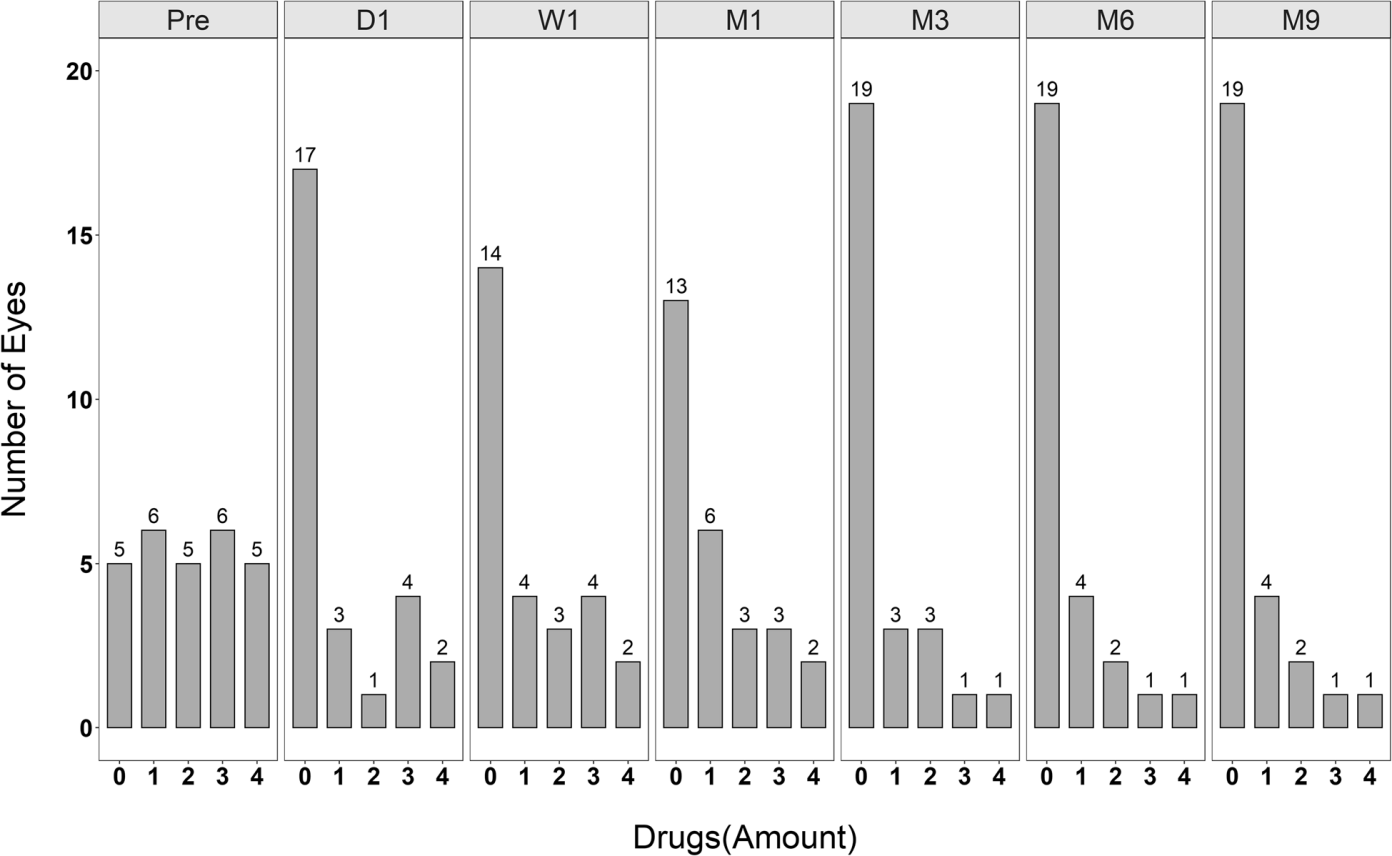
Medication Outcomes. The number of postoperative glaucoma medication was significantly reduced

At 9 months, preoperative glaucoma medication was 1.9 [± 1.41] and postoperatively was 0.56 [± 1.05]. The number of postoperative glaucoma medication was significantly reduced; 19 (70.3%) eyes were without glaucoma medication, 4 (14.8%) eyes with 1 medication, 4 (7.4%) eyes with 2 medications, 1 (3.7%) eye with 3 medications and 1 (3.7%) eye with 4 medications at 9 months (*P* < 0.001). (Fig. 5).

#### Visual acuity outcomes

Preoperative best corrected visual acuity (BCVA) showed and improvement from 0.4 ± 0.4 LogMAR to 0.2 ± 0.4 LogMAR at 9 months with no statistically significant differences (*p* > 0.05).

#### Postoperative complications

The most common complications included: 1) hyphema (66,7%), found during week 1 and resolved spontaneously in all cases at 1 month of follow up 2) Intraoperative reflux bleeding was observed in all cases, but stopped by the time the surgery was completed 3) corneal edema (7.4%), 92,6% with no complications at 1 month 4) Transient hypotony (3,7%) 5) Temporary IOP spike (6%). At 3 months, no complication were reported. Complications such as iris injury, corneal descompensation, corneal injury, cyclodialysis, choroidal hemorrhage or endophthalmitis were not found.

## Discussion

This retrospective study of patients with uncontrolled POAG at different stages of the disease showed a reduction of IOP and glaucoma medication with stable, best corrected visual acuity at 9 month follow-ups with combined, minimally-invasive procedures consisting of phaco, ab-interno trabeculectomy and ECP.

Cataract extraction as a stand alone procedure does not provide a significant reduction of IOP in patients with primary open angle glaucoma [1], therefore a combined treatment with glaucoma surgery is almost always the choice for adequate control of the intraocular pressure. The available evidence suggests at most a modest reduction in IOP from cataract extraction around 1.5–3 mmHg [8] possibly via decompression or mechanical stretch of the TM and Schlemm’s canal [7]. Siegel et al. demonstrated a statistically significant difference between the combined phaco-ECP group versus phaco alone [9].

The combination of ECP with phacoemulsification and ab-interno trabeculectomy with phacoemulsification has been studied [10]. Kaplowitz K. et al. described that ECP used for POAG decreases IOP by 8–47%, to a final average near 15 mmHg [11]. Clement et al. combined phaco-ECP and observed IOP reductions up to 69%, the mean reduction of 23.9% 12 months after treatment [7, 12].

A trial by Berke et al. compared 626 eyes treated with phacoemulsification-ECP, where 81 eyes underwent phacoemulsification alone. The follow-up period ranged from 6 months to 5.5 years. In the phacoemulsification-ECP group, mean IOP decreased 3.4 mmHg, from 19.1 to 15.7 mmHg. In the control group, mean IOP increased 0.7 mmHg, from 18.9 to 18.2 mmHg. More significantly, the number of preoperative glaucoma medications decreased from a mean of 1.53 to 0.65 at the end of the follow-up period in the phacoemulsification-ECP group. There was no visual loss or significant adverse events postoperatively [6]. In our study, the preoperative best corrected visual acuity (BCVA) showed an improvement from preoperative value of 0.4[± 0.4] LogMAR to 0.2 [± 0.4] LogMAR at 9 months with no statistically significant differences.

In our combined study, the mean IOP was reduced 32.9% from baseline and the qualified success was 92.6% at 9 months. Morales et al., with Phaco-ECP, reported the results obtained for IOP lowering to 15 mmHg and report an absolute success of 11.9% with a qualified success of 72.3% [13]. A retrospective Brazilian study on 247 patients, defined success based on IOP 21 mmHg with 3 years of follow-up reported the corresponding rates were 55.7% for absolute success and 90.7% for qualified success [14].

The Kahook dual blade shows promise as a refined and economical device for the treatment of glaucoma [3]. Salinas et al. studied 53 eyes and the mean IOP decreased from 18.4 ± 6.1 mmHg at baseline to 13.9 ± 3.5 mmHg at 6 month follow-ups (23.9% reduction, *p* < 0.001); At 6 months, 63.5% achieved an IOP ≤14 mmHg and the mean number of glaucoma medications was reduced 1.2 ± 1.3 compared to baseline (*P* < 0.001), a reduction of 36.6% was found [15]. With the combined procedures used in ours study, the mean IOP decreased from 17.0 ± 3.7 mmHg at baseline to 11.6 ± 1.9 mmHg at 6 months (31.7% reduction, *P* < 0.001), and an IOP of 11.4 ± 1.8 mmHg at 9 months (32.9% reduction, *P <* 0.001). The IOP was significantly reduced (*P <* 0.001). The mean number of glaucoma medications used was reduced from 1.90 ± 1.41 at baseline pre-op to 0.56 ± 1.05 (29.5% reduction) at 9 months, with statistically significant improvement (*P <* 0.001).

In a preclinical study of human donor of corneo-scleral rims; SooHoo et al. used the dual blade device to incise TM and compare with trabectome. Dual blade showed more complete TM tissue removal with no significant damage to adjacent tissues [3]. Seibol et al., a laboratory evaluation in a human eye perfusion model demonstrated that trabectome treatment across 117.5 ± 12.6 degrees resulted in a decrease of IOP from 18.8 ± 1.7 mmHg to 11.3 ± 1.0 mmHg (*P* < .01) and with dual blade device treatment across 157.5 ± 26.3 degrees, resulted in a decrease of IOP from 18.3 ± 3.0 mmHg to 11.0 ± 2.2 mmHg (*p* < 0.01). The novel dual blade device demonstrated a more complete removal of TM without residual TM leaflets or damage to surrounding tissues and significantly reduced IOP^17^.

Kaplowitz et al., showed that ab-interno trabeculectomy can be expected to lower the IOP by approximately 36% to a final mean IOP around 16 mmHg while decreasing the number of medications by less than one [11]. Fallano et al. used a combined treatment of phaco-trabectome with 18% reduction in IOP^2^. The cases of Trabectome combined with phacoemulsification showed a decrease in IOP from a preoperative mean of 20.0 ± 6.2 mmHg to a mean of 15.9 ± 3.3 mmHg at 12 months (*n* = 45), a decrease of 18% [3]. Francis et al. studied 304 patients treated with combined phaco-trabectome surgery; the mean IOP decrease from 20.0 ± 6.3 mmHg to 14.8 ± 3.5 mmHg at 6 months and 15.5 ± 2.9 mmHg at 1 year. The mean number of glaucoma medications was reduced from 2.65 ± 1.13 to 1.76 ± 1.25 at 6 months and 1.44 ± 1.29 at 1 year [1].

Dang et al. combined trabectome and phaco-trabectome and divided the patients into 4 groups depending on glaucoma severity. The group with the higher glaucoma severity index (GI) had an IOP reduction of 2.34 ± 0.19 mmHg more than the group with lower glaucoma severity index [16]. In our study, all subcategories showed a significant reduction in IOP 9 months after combined surgery. The IOP of the mild glaucoma group was reduced from 18.9 to 11.8 mmHg, from 15.3 to 11.1 mmHg in the moderate group and from 17.4 to 10.8 mmHg in the advanced group.

The review and meta-analysis of Phaco-ECP from Kaplowitz et al., showed the most common complication was hyphema, similar to the present study, and the second most common complication was peripheral anterior synechiae in 24% of the patients. The most serious complication was hypotony 0.09% of all reposted cases [11]. With the use of Trabectome, SooHoo et al. found that all patients with transitory hyphema resolved after 6.4 days [3]. In our study, the most common complications included: hyphema 18 (66.7%), intraoperative blending (observed in all cases), corneal edema (7.4%), transient hypotony (3,7%) and temporary IOP spike (6%) with no vision sequelae and no reoperations.

The weakness of the study were: the retrospective nature, low sample study and lack of control group.

## Conclusion

Cataract extraction with phacoemulsification combined with ab interno trabeculectomy and endoscopic cyclophotocoagulation effectively reduced IOP and dependence on glaucoma medications and showed high safety profile and stable best corrected visual acuity in patients with uncontrolled open angle glaucoma. Further study is needed to compare this combination treatment with the gold standard filtration surgery.

## Data Availability

The datasets used and analysed during this study are available via the corresponding author at reasonable request.

## Abbreviations

BCVA: Best Correted Visual Acuity
CCC: Continuous Curvilinear Capsulorhexis
ECP: Endoscopic cyclophotocoagulation
IOP: Intraocular pressure
IOL: Intraocular Lens
KDB: Kahook Dual Blade
LogMAR: Logarithm of the mínimum angle of resolution
MIGS: Minimally Invasive Glaucoma Surgery
OVD: Ophthalmic Viscosurgical Device
POAG: Primary Open-angle Glaucoma
PHACO: Phacoemulsificiation
SdOCT: Spectral Domain Optical Coherence Tomography
TM: Trabecular Meshwork

## References

[1] Francis BA, Minckler D, Dustin L (2008). Combined cataract extraction and trabeculotomy by the internal approach for coexisting cataract and open-angle glaucoma: initial results. J Cataract Refract Surg.

[2] Fallano K, Bussel I, Kagemann L, Lathrop KL, Loewen N. Training strategies and outcomes of ab interno trabeculectomy with the trabectome. F1000Res. 2017;6. 10.12688/f1000research.10236.2. 10.12688/f1000research.10236.1 PMC5428488 28529695

[3] SooHoo JR, Seibold LK, Kahook MY (2015). Ab Interno trabeculectomy in the adult patient. Middle East Afr J Ophthalmol.

[4] Tanito M (2018). Microhook ab interno trabeculotomy, a novel minimally invasive glaucoma surgery. Clin Ophthalmol.

[5] Wang C, Dang Y, Waxman S, Xia X, Weinreb RN, Loewen NA (2017). Angle stability and outflow in dual blade ab interno trabeculectomy with active versus passive chamber management. PLoS One.

[6] Clement CI, Kampougeris G, Ahmed F, Cordeiro MF, Bloom PA (2013). Combining phacoemulsification with endoscopic cyclophotocoagulation to manage cataract and glaucoma. Clin Exp Ophthalmol.

[7] Walland MJ, Parikh RS, Thomas R (2012). There is insufficient evidence to recommend lens extraction as a treatment for primary open-angle glaucoma: an evidence-based perspective. Clin Exp Ophthalmol.

[8] Siegel MJ, Boling WS, Faridi OS (2015). Combined endoscopic cyclophotocoagulation and phacoemulsification versus phacoemulsification alone in the treatment of mild to moderate glaucoma. Clin Exp Ophthalmol.

[9] Lindfield D, Ritchie RW, Griffiths MF. “Phaco-ECP”: combined endoscopic cyclophotocoagulation and cataract surgery to augment medical control of glaucoma. BMJ Open. 2012;2(3). 10.1136/bmjopen-2011-000578. 10.1136/bmjopen-2011-000578 PMC3367146 22649172

[10] Kaplowitz K, Bussel II, Honkanen R, Schuman JS, Loewen NA (2016). Review and meta-analysis of ab-interno trabeculectomy outcomes. Br J Ophthalmol.

[11] Rathi S, Radcliffe NM (2017). Combined endocyclophotocoagulation and phacoemulsification in the management of moderate glaucoma. Surv Ophthalmol.

[12] Morales J, Al Qahtani M, Khandekar R (2015). Intraocular pressure following phacoemulsification and endoscopic Cyclophotocoagulation for advanced Glaucoma: 1-year outcomes. J Glaucoma.

[13] Lima FEL, de CDM, de AMP (2010). Phacoemulsification and endoscopic cyclophotocoagulation as primary surgical procedure in coexisting cataract and glaucoma. Arq Bras Oftalmol.

[14] Salinas L, Chaudhary A, Berdahl JP, et al. Goniotomy using the Kahook dual blade in severe and refractory Glaucoma: six month outcomes. J Glaucoma. 2018. 10.1097/IJG.0000000000001019. 10.1097/IJG.0000000000001019 29979337

[15] Seibold LK, Soohoo JR, Ammar DA, Kahook MY (2013). Preclinical investigation of ab interno trabeculectomy using a novel dual-blade device. Am J Ophthalmol.

[16] Dang Y, Roy P, Bussel II, Loewen RT, Parikh H, Loewen NA (2016). Combined analysis of trabectome and phaco-trabectome outcomes by glaucoma severity. F1000Res.

